# Addition of vegetable oil to enhance the biosynthesis of butenyl-spinosyn in a high production strain *Saccharopolyspora pogona*

**DOI:** 10.1371/journal.pone.0319332

**Published:** 2025-03-26

**Authors:** Zhouqin Xu, Xinying Li, Chao Guo, Chang Su, Chao Wang

**Affiliations:** 1 Academy of National Food and Strategic Reserves Administration, Institute of Cereal & Oil Science and Technology, Beijing, China; 2 Key Laboratory of Medical Molecule Science and Pharmaceutics Engineering, Ministry of Industry and Information Technology, Institute of Biochemical Engineering, School of Chemistry and Chemical Engineering, Beijing Institute of Technology, Beijing, China; Center for Research and Technology Transfer, VIET NAM

## Abstract

Butenyl-spinosyn discovered from *Saccharopolyspora pogona*, is a broad-spectrum bioinsecticide. In order to further improve the production, the fermentation medium of a high-production strain *Sa. pogona* ASAGF30A11 obtained by mutagenesis, was optimized by adding different species and concentrations vegetable oil. In our study, the effect of peanut oil on the growth and production was proved by monitoring the growth curves, key gene transcription level and content of acyl-CoA. After adding 10 g/L of peanut oil, the additional carbon sources redirected the carbon flux toward strain growth, inhibiting the synthesis of butenyl-spinosyn, while increasing biomass by approximately 1.5-fold. However, when adding 1 g/L of peanut oil, it functions as a surfactant, greatly promoting the synthesis of butenyl-spinosyn, resulting in a 1.52-fold increase in production. The research provides a promising strategy to improve butenyl-spinosyn production.

## 1. Introduction

**Fig 1:**
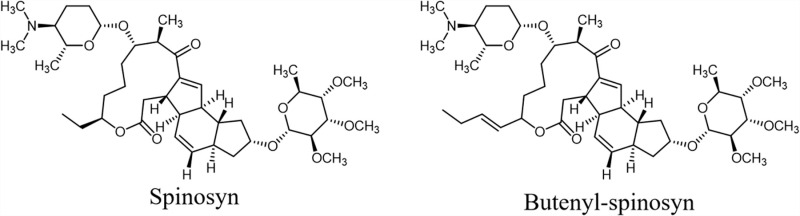
The structural formula of spinosyn and butenyl-spinosyn.

Butenyl-spinosyn, discovered from *Saccharopolyspora pogona*, is a 26-membered ring macrolide with broad-spectrum insecticidal activities [1,2]. The structure of butenyl-spinosyn is very similar to that of spinosyn, containing a macrocyclic structure from malonyl-CoAs, methylmalonyl-CoA and propionyl-CoA, with a forosamine and a tri-O-methylrhamnose, and differs only in a single moiety located at the carbon C21 position (Fig 1) [3]. Compared with spinosyn, butenyl-spinosyn exhibits a broader insecticidal spectrum and increased insecticidal activity. Thus, butenyl-spinosyn demonstrates significant potential for development as an environmentally-friendly insecticide. Many genetic engineering strategies have been employed to improve production, such as the elimination competing pathways, overexpression structure genes and precursor biosynthetic pathways, and regulation of metabolic networks by regulators [4–11]. Metabolic pathways interact with each other, forming a complex metabolic network. Many studies have sought to elucidate regulation mechanisms and enhance production using advanced omics-based technologies [12–16].

Mutagenesis and medium optimization are simple methods for improving the production without straightforward the metabolic network. Increasing mutation rate and evolutionary rates via physical, chemical, and composite mutagenesis facilitates obtaining high-producing, stress-resilient mutant strain [17–19]. Production during microbial fermentation can be further enhanced by optimizing the culture medium composition and fermentation conditions. Carbon source serves as the major source for energy and precursor with its type and concentration influencing the efficiency of secondary metabolites biosynthesis, transport and catabolism. Glucose is a carbon source preferred by most microorganisms, and some other carbon sources, such as fatty acid, may be added later to promote product synthesis. Studies have indicated that fatty acids play essential role in the polyketide synthesis. Adding vegetable oil to the fermentation medium can prevent the carbon catabolite repression (CCR) and improve polyketide production. Fatty acids provide precursor acyl-CoA for polyketide synthesis through the β-oxidative pathway, reducing acyl-CoA consumption by inhibiting fatty acid synthesis. Oil Supplementation enhances the production of monensin and rapamycin [20,21]. When 20 g/L of olive oil was used, the production of lincomycin was increased by 2-fold [22]. Additionally, strain shows varying fatty acid preferences. *Saccharomonospora azurea* prefers primycin production with 4.5 g/L stearic acid [23]. Saturated fatty acids, such as palmitic acid, stearic acid and palmitic acid, significantly promote the synthesis of natamycin and spinosyn [24–26]. Fatty acids can act as surfactant increasing cell membranes permeability. Additionally, fatty acids can serve as extraction solvents for two-phase fermentation. When Tween-80 (as a surfactant) and soybean oil (as an in situ extractant) were supplied at the logarithmic growth phase of fermentation, antrodin C production was improved by 3.6-fold [27]. The adding soybean oil to the fermentation medium as both a carbon source and extraction solvent significantly increased the prodigiosin production [28]. Furthermore, fatty acids can be used as antifoaming agents, enhancing the fermentation stability and efficiency [29–31]. However, the effect of vegetable oil on *Sa. pogona* has not been reported.

In this study, the effects of vegetable oils on a high production strain, ASAGF30A11 derived by mutagenesis, were examined. Specifically, the types and concentrations of additional vegetable oils on production and growth were investigated. The observation that 1 g/L vegetable oils dramatically enhanced production, while 10 g/L oils increased the biomass, was analyzed by monitoring the growth curves, key gene transcription level and content of acyl-CoA. Finally, a potential molecular mechanism was proposed based on these finding.

## 2. Materials and methods

### 2.1. Strain, medium, and culture conditions

*Sa. pogona* ASAGF30A11 (butenyl-spinosyn high-production strain; generated by ^60^Co γ-ray and Nitrosoguanidine (NTG) mutagenesis) was stored as a glycerol freezer stock at -80°C in our laboratory. Spores were cultivated on GYM agar plates (glucose 4 g/L, yeast extract 4 g/L, CaCO_3_ 2 g/L, agar 20 g/L) for 7-10 days for sporulation. Fresh spores were then collected and cultured in seed medium (TSB, pH=7.2) for 48 h. A 10% (vol/vol) seed culture was subsequently used to inoculate fermentation medium (glucose 60 g/L, soluble starch 20 g/L, corn steep solid 10 g/L, peptone milk 20 g/L, CaCO_3_ 5 g/L, MgSO_4_·7H_2_O 1 g/L, NaCl 1 g/L, pH 7.2), and cultivated at 30°C and 200 r/min for 7 days. Vegetable oil was purchased from the supermarket (S1 Table). All experiments were performed in triplicate in 3 batches.

### 2.2. Analysis of biomass, productivity, glucose consumption, residual oil and acyl-CoA

Biomass measure was calculated using the wet weight method. An empty centrifuge tube (10 mL) was weighed and recorded as M1. The fermentation broth was then added to the tube, and its weight recorded as M2. The sample was centrifuged, and the sediment weight in the tube was recorded as M3. The analytical balance used in this study has an accuracy of ±0.1 mg. The biomass=(M3-M1)/(M2-M1) × 100% [16].

For butenyl-spinosyn production, fermentation broth was extracted with three volumes of methanol, followed by 30 minutes of ultrasonication. The extracts were then centrifuged at 12,000 r/min for 10 min. Waters 2998 HPLC system equipped with an Agilent reversed-phase TC-C18 column (100×4.6 mm, 3.5 μm) was used to detect the production levels. The isocratic mobile phase consisted of 45% methanol, 45% acetonitrile, and 10% water containing 0.05% ammonium acetate. The samples were analyzed at a flow rate of 1.0 mL/min for 10 min at a UV detector detection wavelength of 244 nm.

Residual glucose concentration in the fermentation broth was measured using SBA-40E biosensor analyzer (Biology Institute of Shandong Academy of Sciences, China) following the manufacturer’s instructions.

The residual oils were quantified by extracting the fermentation broth with modifications based on a previously described method [32]. Residual oils were measured by extracting the fermentation broth with petroleum ether, followed by solvent evaporation.

1 mL of fermentation broth was collected and centrifuged at 12,000 r/min for 10 min. The precipitate was resuspended in PBS, and the supernatant was used for following 5 minutes of sonication. Acyl-CoA was measured using commercial enzyme-linked immunosorbent assay (ELISA) kits (Shanghai Enzyme-linked Biotechnology Co., Ltd., China).

### 2.3. Gene expression analysis by quantitative real-time PCR (qRT-PCR)

*Sa. pogona* cells were harvested on the 3rd, 5th and 7th days, and centrifuged at 12,000 r/min at 4°C. Total RNA was extracted from *Sa. pogona* cells using the RNAprep Pure Cell/Bacteria kit (Tiangen, China). The extracted RNA was reverse transcribed to cDNA using the First Strand cDNA Maxima Synthesis kit (TOYOBO, Shanghai, China). Real time qPCR amplification was performed using Power SYBR Green PCR Master Mix (Thermo Fisher Scientific, Waltham, USA) following previously documented procedures. Target gene expression was quantified using the 2^−ΔΔCT^ method. Relative gene expression levels were normalized against the expression of the 16S rRNA gene. The primers used in qRT-PCR were listed in S2 Table.

### 2.4. Statistical analysis

Using SPSS 26.0 and Origin 2021 software to process data, each experiment was set up with 3 replicates. Statistical significance was analyzed by Student’s t-test (two-tail) with ***p < 0.001, **p < 0.01, and *p < 0.05.

## 3. Results

### 3.1. Effects of vegetable oil on *Sa. pogona*

**Fig 2:**
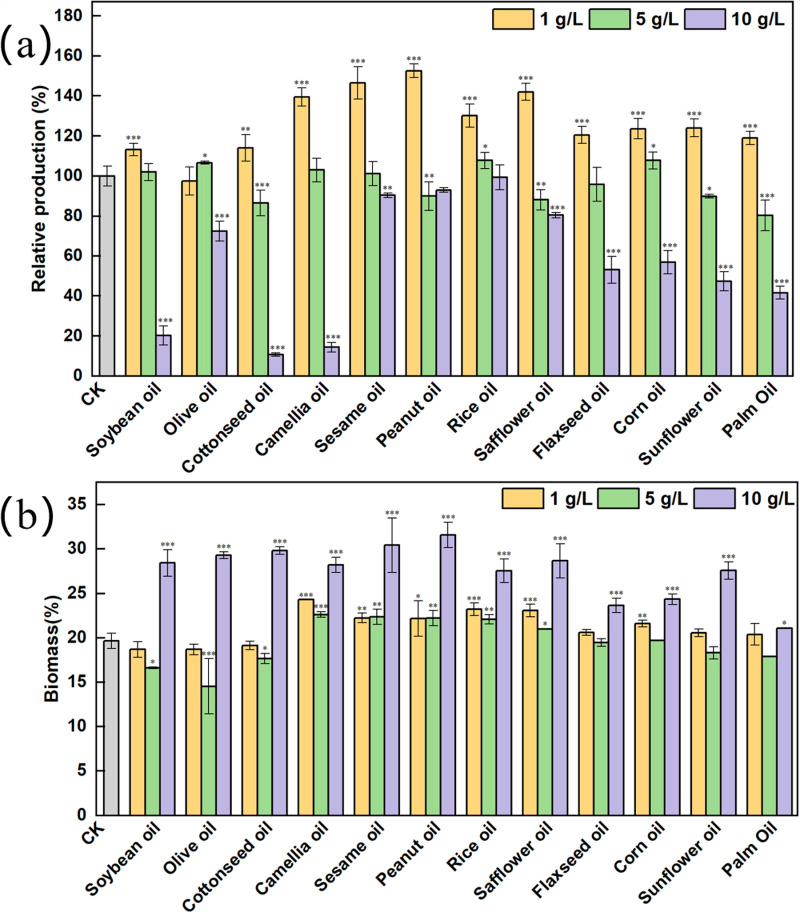
Effect of various vegetable oils on *Sa.* ***pogona*.** The effects on butenyl-spinosyn production (a) and biomass (b) were quantitatively analyzed. The control group is *Sa. pogona* ASAGF30A11 (a high production strain of butenyl-spinosyn; generated by 60Co γ-ray and NTG mutagenesis).

The high production strain *Sa. pogona* ASAGF30A11, obtained by ^60^Co γ-ray and NTG mutagenesis in previous studies, exhibited butenyl-spinosyn production approximately 3-fold higher than that of wild type ASAGF58. To further enhance the production, exogenous vegetable oils were added directly to the fermentation medium. Conventional vegetable oil, including soybean oil, olive oil, cottonseed oil, camellia oil, sesame oil, peanut oil, rice oil, safflower oil, flaxseed oil, corn oil, sunflower oil, and palm oil, were selected for the experimental group, while control was without vegetable oil. After a 7-day shake-flask culture, it was observed that adding 1 g/L peanut oil increased the production by 1.52-fold, followed by sesame oil, safflower oil and camellia oil, which increased by 1.46-, 1.42- and 1.4-fold, respectively. The lowest production was observed with the addition of 1 g/L olive oil. The other vegetable oils (soybean oil, cottonseed oil, flaxseed oil, corn oil, sunflower oil, palm oil, soybean oil and sunflower oil) increased the production by only 0-25% (Fig 2a). Biomass did not change significantly, indicating that the increase in butenyl-spinosyn production was not due to biomass increase.

To further enhance production, the effects of varying vegetable oil concentrations on fermentation were investigated. At a concentration of 5 g/L of vegetable oil, the production of butenyl-spinosyn was reduced to control levels, and the biomass levels were similar in fermentation medium containing either 1 g/L or 5 g/L oil. At 10 g/L, peanut oil, sesame oil, safflower oil, and rice oil had no significant effect on production, but the biomass was increased by 1.39-, 1.34-, 1.32-, and 1.22-fold (Fig 2b). Production decreased by 5–25% in fermentation with soybean oil, cottonseed oil, and camellia oil, while other oils led to a 40–80% reduction. Similarly, biomass increased by 20-30% (Fig 2a).

Based on the above results, the production of butenyl-spinosyn was significantly improved in medium containing 1 g/L vegetable oil, while the biomass reached its maximum in medium with 10 g/L vegetable oil. Consequently, among all oils tested, peanut oil was demonstrated to be the most efficient in enhancing improving production and biomass in different concentration. Therefore, peanut oil was chosen to investigate the mechanism underlying the effect of vegetable oil on *Sa. pogona* production and biomass.

### 3.2. The effect of peanut oil on *Sa. pogona* growth

**Fig 3:**
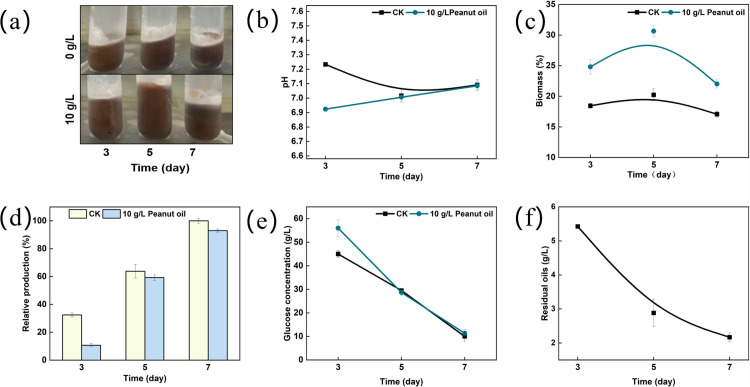
Effect of peanut oil on the growth of *Sa. pogona.* (a) Mycelial and insoluble fraction collected by centrifugation; Biomass of *Sa. pogona* (b), Butenyl-spinosyn production (c), Glucose concentration (d), Residual peanut oil (e), and pH changes (f) between the control and experimental groups with 10 g/L peanut oil on the 3rd, 5th, and 7th days.

Based on the results above, fermentation with the addition of 10 g/L peanut oil was performed for 7 days, with fermentation without oil serving as the control. The biomass was measured on the 3rd, 5th and 7th days to investigate the effect of peanut oil on growth. As shown in Fig 3a, adding 10 g/L peanut oil significantly increased biomass, especially on the 5th day. The growth rate with oil was highest on the 3rd day, while it only was only 1.3%/day in the control. The growth rate started to decrease after the 3rd day, but the biomass reached its maximum (30%), which was 1.5-fold higher than that in the control on the 5th day (Fig 3b). Meanwhile, the production was lower than the control on the 3rd day, but reached a comparable level by the 7th day (Fig 3c). Notably, (I) adding 10 g/L peanut oil significantly reduced the production capacity per cell, (II) all significant differences were observed on the 3rd day. To verify this hypothesis, the glucose and peanut oil consumption were monitored. On the 3rd day, 5 g/L glucose and 4.5 g/L peanut oil were consumed, while 15 g/L glucose was consumed in the control. There was no difference in glucose consumption on the 5th and 7th day, but 2.5 g/L and 0.5 g/L oil were consumed, respectively (Figs 3d and 3e). A change in pH was observed on the 3rd day with pH lower than that of the control. Hydrolysis of peanut oil may produce organic acids, decreasing the medium’s pH to 6.9. As oil consumption continued, pH returned to the control level (Fig 3f). Collectively, these results suggest that the addition of the 10 g/L vegetable oils served as a carbon source enhancing growth-related pathways.

**Fig 4:**
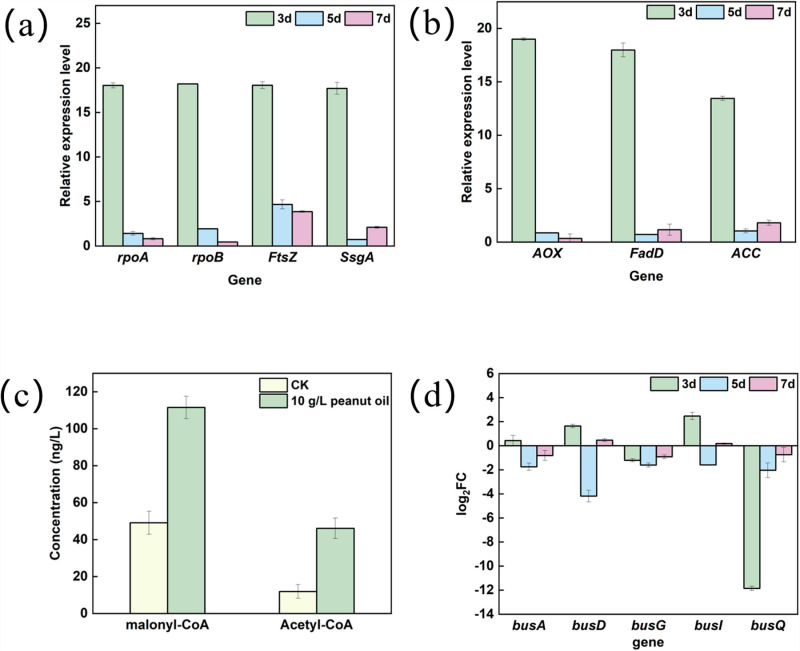
Relative gene expression level and concentration of acyl-CoA. Relative expression levels of growth-related genes (a)and fatty acid metabolic pathway genes(b); (c) Comparison of intracellular acyl-CoA concentrations; (d) Relative expression levels of genes involved in butenyl-spinosyn biosynthesis on the 3rd, 5th and 7th days.

The expression level of growth-related genes on the 3rd, 5th, and 7th days, were monitored using qRT-PCR. Morphologically differentiation-related genes *rpoA*, *rpoB*, *ftsZ* and *ssgA* were upregulated by 18.03-, 18.20-, 17.70- and 18.05-fold on the 3rd day, consistent with the observed increase in biomass due to peanut oil addition (Fig 4a). Acyl-CoA is a key precursor in the polyketide chain. The transcription level of gene ACOX gene (acyl-CoA oxidase) and *fadD* (acyl-CoA synthetase), involved in the β-oxidative pathway were significantly upregulated by 17.98- and 19-fold compared to the control. Additionally, the transcription of *acc* (acetyl-CoA carboxylase), responsible for the synthesis of malonyl-CoA, was upregulated by 13.45- fold (Fig 4b). Concurrently, the concentration of acetyl-CoA and malonyl-CoA were increased by 5.6- and 2.6-fold on the 3rd day (Fig 4c). The transcription level of gene involved in butenyl-spinosyn biosynthesis (*busA*, *busD*, *busG*, *busI* and *busQ*) was downregulated, resulting in a decreased synthesizing capacity (Fig 4d). Notably, most vegetable oils at a concentration of 10 g/L could increase the biomass. These results further demonstrated that the addition of 10 g/L vegetable oils served as a carbon source for strain growth. The main components of vegetable oils likely contribute to the observed differences in biomass (Table S2).

### 3.3 The effect of peanut oil on the production of butenyl-spinosyn

**Fig 5:**
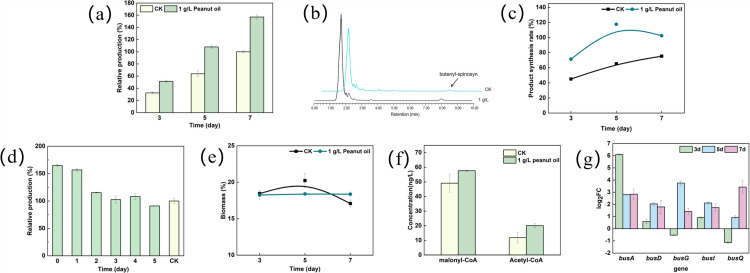
Effect of 1 g/L peanut oil on the production of butenyl-spinosyn. (a) Relative production on the 3rd, 5th and 7th days when adding oil at the start of fermentation; (b) The HPLC profiles of butenyl-spinosyn. The detection wavelength was set at 244 nm and the chromatographic peak of butenyl-spinosyn appeared at 7.9 min; (c) Synthesis rate of butenyl-spinosyn; (d) Relative production of butenyl-spinosyn with oil added at different times; (e) The biomass of *Sa. pogona*; (f) Intracellular malonyl-CoA level comparison; (g) Transcription levels of butenyl-spinosyn synthesis genes.

Studies have shown that fatty acids can promote polyketide biosynthesis. However, in our studies, low concentrations of vegetable oils promoted the synthesis of butenyl-spinosyn. To confirm the effect of peanut oil on butenyl-spinosyn production, production levels were measured on the 3rd, 5th and 7th days. The production was 40%, 45%, and 50% higher than that in control, respectively (Figs 5a and 5b). The synthesis rate was higher than the control and peaked on the 5th day (Fig 5b). Subsequently, the optimal time for peanut oil addition was tested. 1 g/L peanut oil was added to the medium at 0, 24, 48, 72, 96 and 120 h during the fermentation to study the effect of the addition timing on production. As shown in Fig 5c, the production of butenyl-spinosyn was highest when oil was added at the start of fermentation. The adding time variation on the production showed a gradual downward trend after 48 h, and there was no effect when added during later fermentation stages. The biomass showed no significant difference when adding 1 g/L oil (Fig 5d).

In fact, 1 g/L vegetable oil was not sufficient to serve as a carbon source. The concentrations of malonyl-CoA and acetyl-CoA showed no significant difference between the control and experiment group (Fig 5e). The transcription levels of *busA*, *busD*, *busG*, *busI* and *busQ* involved in butenyl-spinosyn synthesis were significantly upregulated compared to the control (Fig 5f). Overall, the addition of 1 g/L peanut oil did not affect the biomass, but improved the synthesis rate. The minimal change in acyl-CoA concentration suggested that 1 g/L peanut oil redirected the metabolic flux toward the synthesis of butenyl-spinosyn.

**Fig 6:**
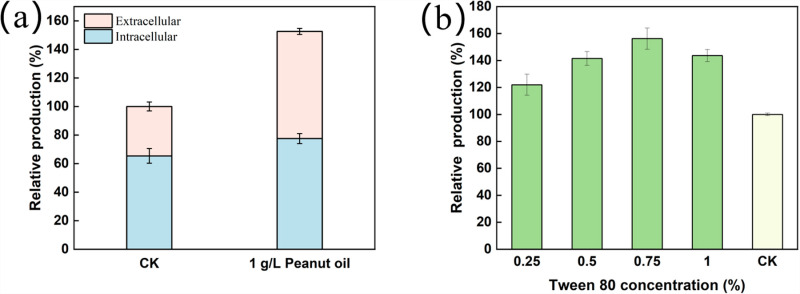
Mechanism of production enhancement by the addition 1 g/L peanut oil. (a) Ratio of extracellular to intracellular butenyl-spinosyn; (b) Effect of Tween 80 on the production of butenyl-spinosyn.

Vegetable oil can serve as a carbon source, anti-foaming agent and surfactant during fermentation. Initially, the roles of carbon source and anti-foaming agent were excluded due to the low addition and limited fermentation volume. To further verify the function of 1 g/L vegetable oil, the levels of extracellular and intracellular butenyl-spinosyn were measured on the 7th day. The ratio of extracellular to intracellular butenyl-spinosyn approximately 1:1 with 1 g/L peanut oil, higher than the 0.5:1 ratio observed without oil addition (Fig 6a). This result suggested that 1 g/L oil may act as a surfactant to promote product efflux. The efflux of butenyl-spinosyn reduced cellular stress, thereby promoting further product synthesis.

To confirm that adding a surfactant promotes the synthesis of butenyl-spinosyn, other surfactants were tested in the medium. The production of butenyl-spinosyn was enhanced by the addition of Tween 80 at concentrations ranging from 0% to 1%. At a concentration of 0.75%, the production reached its peak, 1.5-fold higher than the control (Fig 6b). The effects of peanut oil and Tween 80 were comparable.

**Fig 7:**
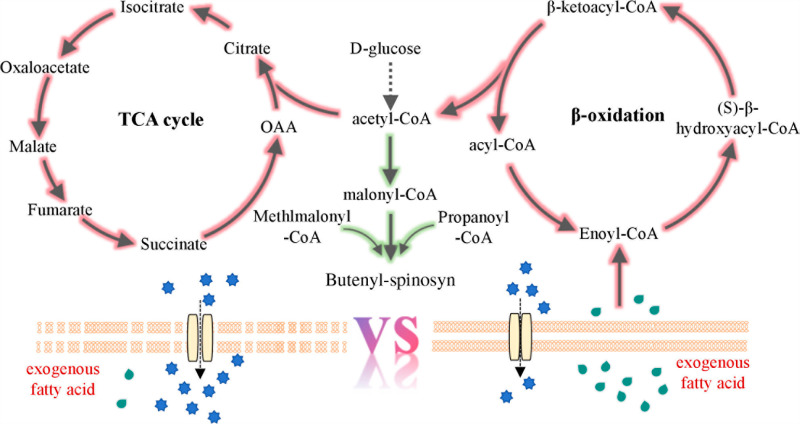
The mechanism of vegetable oil on *Sa. pogona.*

In summary, the effects of species and concentrations of vegetable oils on butenyl-spinosyn production were investigated. The addition of 10 g/L vegetable oil improved the biomass but not production, and the precursor and energy obtained from the fatty acid β-oxidation pathway were primarily utilized for cell growth. 1 g/L peanut oil could be used as surfactant to improve the production of butenyl-spinosyn by 1.5-fold. However, it did not affect the growth (Fig 7). This study represents the first report on improving butenyl-spinosyn production by adding vegetable oil as a surfactant and provides a reference for improving production in the further.

## 4. Discussion

Triglycerides, the primary components of vegetable oils, play an important fermentation role. They can be used as a carbon source and promote the production synthesis under glucose repression. It has been reported that adding 20-30 g/L oil can improve the production of polyketide. In strain *Sa. pogona*, the addition of over 15 g/L of vegetable oil adversely affected normal growth. It is speculated that vegetable oil may disrupt the normal function of the cell membrane or the generation of H_2_O_2_ may have a deleterious effect on the growth [26]. The maximum acceptable oil concentration for growth was 10 g/L. Secondary metabolites are not essential for the survival of microorganisms, however, they play an important role in the interaction between microorganisms and the environment, promoting adaptation to both biotic and abiotic stress [33]. The addition of 10 g/L of peanut oil promoted an increase in biomass while reducing the synthesis of secondary metabolites, indicating that the vegetable oil medium was rich in nutrients, met the conditions required for the growth of the strain and was suitable for enhancing biomass production.

Given that the concentration of peanut oil is only 1 g/L, it is speculated that it may function as a surfactant. Surfactants can alter the composition of the cell membrane, thereby increasing the permeability, which facilitates the efflux of secondary metabolites from the cells, reduces the detrimental effects of these metabolites on cellular function, and enhances synthesis [34]. Triglycerides, are comprised of different types of fatty acids. The strain may exhibit varying preferences for the saturation and length of fatty acids. Therefore, other vegetable oils exhibited varying effects on production (Fig 2a and S3 Table). Studies have shown that vegetable oil contain trace elements, which also have positive effect on production [35].

Vegetable oil can not only serve as a carbon source and surfactant, but it may also enhance the production of butenyl-spinosyn based fungicides by altering the surface tension of the culture medium, increasing the dissolved oxygen levels or incorporating defoamers [30,36]. This requires further verification through experiments.

## Supporting information

Table S1 Types and sources of vegetable oil. (XLSX)

Table S2 The primers in the study. (XLSX)

Table S3 Composition of fatty acids in vegetable oil (%). (XLSX)

Table S4 Raw data. (XLSX)
